# Supplementary material for: Machine-Learning-Based Radiomics MRI Model for Survival Prediction of Recurrent Glioblastomas Treated with Bevacizumab
Source: Diagnostics (Basel). 2021 Jul 14;11(7):1263. doi: 10.3390/diagnostics11071263 (PMC8305059; doi:10.3390/diagnostics11071263)
Supplement: Supplementary file 1 [file diagnostics-11-01263-s001.zip › diagnostics-1285294-supplementary.pdf]

Supplementary Materials

Table S1. Cross-validation results (± 1 std deviation) for the different machine-learning algorithms.

ML algorithm	OS 9 months	OS 12 months	OS 15 months	PFS 6 months	PFS 9 months
RFT	0.57 ± 0.09	0.68 ± 0.11	0.63 ± 0.15	0.57 ± 0.05	0.60 ± 0.15
Logistic regression	0.64 ± 0.12	0.68 ± 0.13	0.58 ± 0.13	0.60 ± 0.04	0.60 ± 0.10
Gradient boosting	0.57 ± 0.09	0.69 ± 0.12	0.61 ± 0.07	0.57 ± 0.08	0.59 ± 0.15
Knn	0.63 ± 0.10	0.66 ± 0.14	0.53 ± 0.16	0.52 ± 0.12	0.57 ± 0.13
Naïve Bayes	0.60 ± 0.14	0.67 ± 0.15	0.58 ± 0.09	0.58 ± 0.08	0.59 ± 0.20
Adaboost	0.54 ± 0.13	0.67 ± 0.10	0.60 ± 0.09	0.57 ± 0.09	0.59 ± 0.19
SVM	0.60 ± 0.10	0.70 ± 0.20	0.60 ± 0.11	0.46 ± 0.10	0.64 ± 0.16

Table S2. Results of the binary classification models on PFS.

Classification model	Weighted average precision	Weighted average recall	AUC on test (on train)
PFS 6 months	0.55	0.54	0.56 (0.71)
PFS 9 months	0.66	0.62	0.69 (0.82)

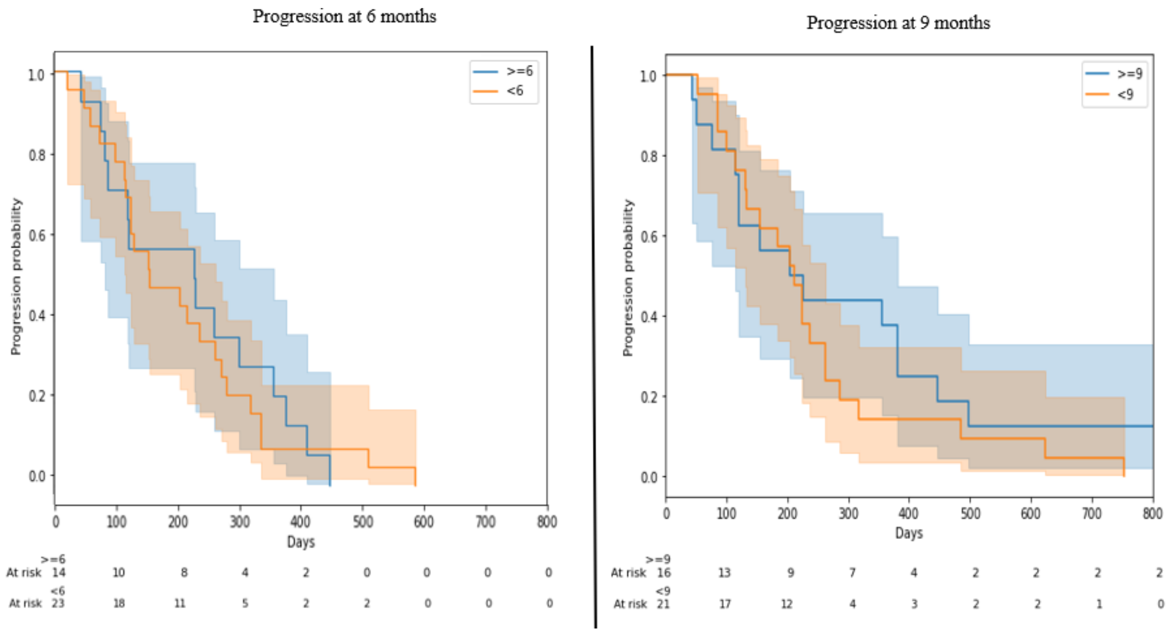

Figure S1. Kaplan–Meier curves on the test sets of the binary classification models on PFS.
